# A Novel *Ex Vivo* Drug Assay for Assessing the Transmission-Blocking Activity of Compounds on Field-Isolated Plasmodium falciparum Gametocytes

**DOI:** 10.1128/aac.01001-22

**Published:** 2022-11-02

**Authors:** Dinkorma T. Ouologuem, Laurent Dembele, Antoine Dara, Aminatou K. Kone, Nouhoum Diallo, Cheick P. O. Sangare, Fatoumata I. Ballo, François Dao, Siaka Goita, Aboubecrin S. Haidara, Aliou Traore, Amadou B. Niangaly, Souleymane Dama, Sekou Sissoko, Fanta Sogore, Jacob N. Dara, Yacouba N. Barre, Amadou Daou, Fatoumata Cisse, Ousmaila Diakite, Diagassan Doumbia, Sekou Koumare, Bakary Fofana, Fatalmoudou Tandina, Daman Sylla, Adama Sacko, Mamadou Coulibaly, Mamadou M. Tekete, Amed Ouattara, Abdoulaye A. Djimde

**Affiliations:** a Malaria Research and Training Center, Faculty of Medicine and Dentistry, Faculty of Pharmacy, University of Sciences, Techniques, and Technologies of Bamako, Bamako, Mali; b Center for Vaccine Development and Global Health, University of Maryland School of Medicine, Baltimore, Maryland, USA

**Keywords:** field-isolated gametocytes, *ex vivo*, culture, drug assay, transmission-blocking activity, direct membrane feeding assay

## Abstract

The discovery and development of transmission-blocking therapies challenge malaria elimination and necessitate standard and reproducible bioassays to measure the blocking properties of antimalarial drugs and candidate compounds. Most of the current bioassays evaluating the transmission-blocking activity of compounds rely on laboratory-adapted *Plasmodium* strains. Transmission-blocking data from clinical gametocyte isolates could help select novel transmission-blocking candidates for further development. Using freshly collected Plasmodium falciparum gametocytes from asymptomatic individuals, we first optimized *ex vivo* culture conditions to improve gametocyte viability and infectiousness by testing several culture parameters. We next pre-exposed *ex vivo* field-isolated gametocytes to chloroquine, dihydroartemisinin, primaquine, KDU691, GNF179, and oryzalin for 48 h prior to direct membrane feeding. We measured the activity of the drug on the ability of gametocytes to resume the sexual life cycle in *Anopheles* after drug exposure. Using 57 blood samples collected from Malian volunteers aged 6 to 15 years, we demonstrate that the infectivity of freshly collected field gametocytes can be preserved and improved *ex vivo* in a culture medium supplemented with 10% horse serum at 4% hematocrit for 48 h. Moreover, our optimized drug assay displays the weak transmission-blocking activity of chloroquine and dihydroartemisinin, while primaquine and oryzalin exhibited a transmission-blocking activity of ~50% at 1 μM. KDU691 and GNF179 both interrupted *Plasmodium* transmission at 1 μM and 5 nM, respectively. This new approach, if implemented, has the potential to accelerate the screening of compounds with transmission-blocking activity.

## INTRODUCTION

The life cycle of the malaria parasite involves two different hosts and many distinct parasite stages, but the gametocyte stages are the target for transmission-blocking therapies (1–3). These sexual stages are the only form of the parasite in the bloodstream of the human host that ensure *Plasmodium* transmission to the *Anopheles* mosquito. They form one of the bottleneck stages in the entire life cycle of the malaria parasite (4). Despite the focused attention on gametocytes for successful malaria eradication, current therapeutics have little or no activity against mature gametocytes. Indeed, artemisinin combination therapies (ACTs) rapidly clear the *Plasmodium* stages responsible for the clinical disease but fail to block parasite transmission (5). 8-Aminoquinoline, primaquine, and tafenoquine have potent gametocytocidal properties and have been shown to block transmission (6–8). However, the use of these drugs is limited by their hemolytic toxicity observed in patients with glucose-6-phosphate dehydrogenase deficiency, a genetic condition with a high prevalence in regions of malaria endemicity (9–11). Extensive research is under way to discover new drugs that can effectively target *Plasmodium* gametocytes (12–14). Newer classes of compounds, including the spiroindolones (KAF246), imidazolopiperazines (GNF179), imidazopyrazines (KDU691), and quinoline-4-carboxamides (DDD107498), have shown potent transmission-blocking activities, and some of their closely related compounds (KAE609 or cipargamine, KAF156) have made it to clinical testing (15–21).

The discovery and development of transmission-blocking therapies do require standard and reproducible bioassays to reliably measure the blocking properties. Several assays are currently available for evaluating the gametocytocidal activity of compounds (1, 22–26). Some of these tools measure parasite metabolites, including *Plasmodium* lactate dehydrogenase activity (pLDH) and the oxidoreduction metabolites (ATP) (22, 23). Others measure stage-specific effects of compounds by detecting male and female gametogenesis markers (24). Most of the above-described experimental tools measure the transmission-blocking activity of compounds mainly *in vitro* and use laboratory-adapted parasites. Hence, the *in vitro* read-out and the use of laboratory-adapted strains may not fully predict the sensitivity of the *Plasmodium* isolates circulating in regions of malaria endemicity. Moreover, most Plasmodium falciparum strains from regions of endemicity have not been adapted in the laboratory for gametocyte production and transmission-blocking activity assessment (27).

Standard membrane feeding assays (SMFA) remain the gold standard approach to measure the transmission-blocking activity and can be adjusted to evaluate the effect of a compound on any plasmodial stages found in the mosquito, including gametocyte viability, gamete, oocyst, and sporozoite development (28, 29). For SMFA, gametocytes from laboratory-adapted strains are produced *in vitro*, subjected to specific experimental conditions, and fed to mosquitoes to determine the ability of the parasite to establish an infection in the mosquito (28). While SMFA closely mimic parasite transmission, this approach fails to capture parasite population diversity seen in the field and to anticipate the susceptibility of clinical isolates to drugs.

Direct membrane feeding assay (DMFA), another variant of the mosquito feeding experiment, uses blood samples from malaria-infected individuals as the source of gametocytes, and the feeding experiment must be conducted immediately or at most a few hours after blood collection (30, 31). While DMFA incorporates the complexity of malaria transmission, as observed in settings of malaria endemicity, the lack of optimized parameters to maintain viable and infectious gametocytes days after blood collections limits their use in the *ex vivo* drug sensitivity assay (32).

In this paper, we report a novel transmission-blocking assay that uses *Plasmodium* gametocytes freshly isolated from naturally infected asymptomatic individuals. The study provides optimized culture conditions to maintain field-isolated viable gametocytes and to enhance parasite infectivity to *Anopheles* mosquitoes. The optimized culture setting was used to establish a novel *ex vivo* drug sensitivity assay by exposing field-isolated gametocytes to reference compounds for 48 h. *Anopheles* mosquitoes were fed with the derived samples to measure the impact of antimalarial drugs and candidate compounds on the ability of gametocyte to resume the sexual life cycle in *Anopheles* after drug exposure.

## RESULTS

### Optimization of the conditions to preserve field-isolated gametocytes viable and infectious.

**FIG 1:**
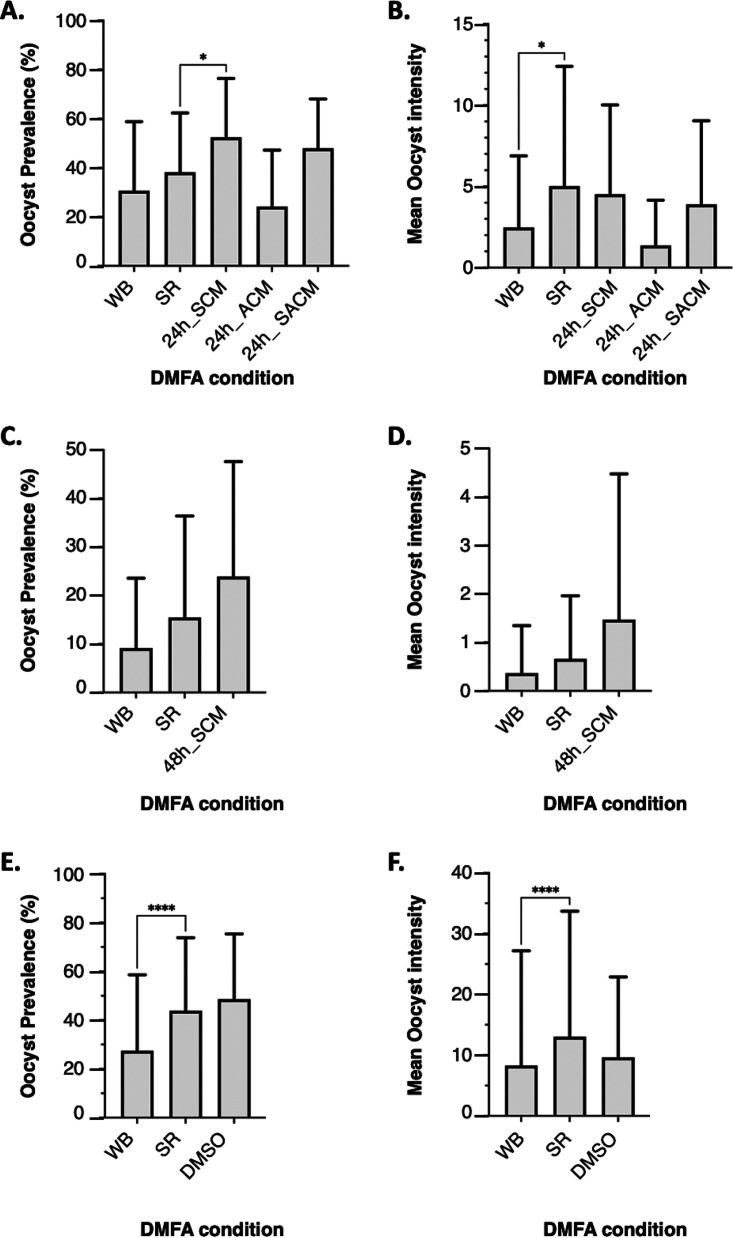
Infectivity of field-isolated gametocytes to Anopheles coluzzii at baseline and following *ex vivo* maintenance under culture conditions in various media for 24 h and 48 h. (A) Oocyst prevalence (number of positive mosquitoes) at baseline (WB and SR) and after a 24-h *ex vivo* exposure under culture condition in SCM, ACM, and SACM. At baseline, the mean oocyst prevalence was 30.6% and 38.2% for WB-DMFA and SR-DMFA, respectively. Compared with SR-DMFA, the mean oocyst prevalence significantly increased to 52.4% when collected gametocytes were maintained in SCM (*P* = 0.03). (B) Oocyst intensity (number of oocysts per mosquitoes) at baseline and after a 24-h *ex vivo* exposure under culture condition in SCM, ACM, and SACM. The mean oocyst intensity was significantly greater in SR-DMFA than that in WB-DMFA (5.02 versus 2.5 oocysts/mosquito, *P* = 0.02), suggesting that the removal of patient serum improved oocyst formation. (C) Oocyst prevalence at baseline and after *ex vivo* exposure under culture condition in SCM for 48 h. Oocyst prevalence from a sample maintained under culture condition is comparable to that of SR samples (23.9 versus 15.5, *P* = 0.1). (D) Oocyst intensity at baseline and after *ex vivo* exposure under culture condition in SCM for 48 h. Oocyst intensity from a sample maintained under culture condition is comparable to that of SR samples (0.6 versus 1.5 oocyst per mosquito *P* = 0.06). (E) Oocyst prevalence at baseline and after *ex vivo* exposure under culture condition in DMSO for 48 h. Infectivity was significantly higher when the mosquitoes were fed with SR samples than that when mosquitoes were fed with WB samples (43.9 versus 27.4; *P* < 10^−4^). Oocyst prevalence from samples maintained in DMSO under culture condition is comparable to that of SR samples (48.6 versus 43.9, *P* = 0.3). (F) Oocyst intensity at baseline and after *ex vivo* exposure under culture condition in DMSO for 48 h. Oocyst intensity was significantly higher in samples from SR-DMFA than that in samples from WB-DMFA (13 versus 8 oocyst per mosquito; *P* < 10^−4^). Oocyst intensity from samples maintained in DMSO under culture condition is comparable to that of SR samples (9.6 versus 13 oocyst per mosquito; *P* = 0.6). Plots represent the mean and the horizontal lines within the histogram indicate the standard deviation. Asterisks indicate significant (*P* < 0.05) differences between groups compared by the Wilcoxon signed-rank test. SCM, serum culture medium; ACM, AlbuMax culture medium; SACM, serum-AlbuMax culture medium; WB, whole blood; SR, serum replacement; DMFA, direct membrane feeding assay.

The first set of experiments was performed in a series of 35 DMFAs using P. falciparum gametocyte-positive blood from 7 donors (Table 1). Gametocyte densities varied among the experimental blood samples from 24 to 384 gametocytes per microliter of blood (Table 1, see Table S2 in the supplemental material). At baseline, the oocyst prevalence was 30.6% and 38.2% for whole-blood DMFA (WB-DMFA) and serum replacement DMFA (SR-DMFA), respectively (Fig. 1A). The mean oocyst intensity was significantly greater in SR-DMFA than that in WB-DMFA (5.02 versus 2.5 oocysts/mosquito, *P* = 0.02), suggesting that the removal of patient serum improved oocyst formation (Fig. 1B). Compared with SR-DMFA, the oocyst prevalence significantly increased to 52.4% when collected gametocytes were maintained in serum culture medium (SCM) (*P* = 0.03) (Fig. 1A). The *ex vivo* condition used in SCM did not affect the number of oocysts per mosquito guts (5.02 versus 4 oocysts/guts for SR and SCM, respectively) (Fig. 1B). The data show that of the three media tested, the SCM was best suited to preserve gametocyte viability and infectivity *ex vivo* at 24 h (*P* = 0.02 and *P* = 0.001 for oocyst density and oocyst intensity, respectively).

**TABLE 1:** Summary of blood sample characteristics, *ex vivo* maintenance conditions, and mosquito feeding experiments^*a*^

*Ex vivo* maintenance condition	Results of *ex vivo* maintenance optimization after:^*b*^		Results of drug assay expt (48 h in SCM) with:^*c*^					
	24 h (no drug)	48 h (no drug)	CQ	DHA	PRQ	KDU691	GNF179	ORY
No. of clinical isolates tested	7	11	7	4	4	6	7	7
No. of mosquitoes in feeding expt	35	33	49	28	28	42	49	49
Gametocyte density (gametocytes/μL)	24–384	16–64	48–1,220	24–1,198	47–1,198	64–3,888	72–2,845	16–822
No. of mosquitoes dissected								
WB-DMFA	335	546	205	88	90	161	155	219
SR-DMFA	322	580	203	93	93	144	181	226
SCM-DMFA	269	416	*	*	*	*	*	*
ACM-DMFA	301	*	*	*	*	*	*	*
SACM-DMFA	282	*	*	*	*	*	*	*
Drug concn^*d*^								
SCM-DMSO	*	*	231	128	57	173	161	203
SCM-Conc 1	*	*	224	120	68	158	148	225
SCM-Conc 2	*	*	226	117	78	192	146	270
SCM-Conc 3	*	*	236	118	73	166	132	236
SCM-Conc 4	*	*	218	122	82	163	156	238

a *, Experiment not performed; SCM, serum culture medium; ACM, AlbuMax culture medium; SACM, serum-AlbuMax culture medium; WB, whole blood; SR, serum replacement; DMFA, direct membrane feeding assay.

b For the optimization of *ex vivo* parameters necessary to ensure field-isolated gametocyte survival and infectivity and to set the ground for subsequent drug screening experiments, we first tested three types of culture medium (SCM, ACM, and SACM). Field gametocytes were maintained *ex vivo* under culture conditions in the different media for 24 h or 48 h prior to feeding experiments. For the 24 h *ex vivo* testing, an aliquot of the blood sample was concomitantly diluted in SCM, SACM, and ACM.

c For the transmission blocking drug assay, we evaluated the transmission-blocking activity of chloroquine (CQ), dihydroartemisinin (DHA), primaquine (PRQ), the *Plasmodium* phosphatidylinositol-4-OH kinase (PI4K)-specific inhibitor KDU691, and the imidazolopiperazine GNF179. There were differences in the concentration range tested for each drug as the drug concentrations were selected based on published IC_50_ data. For the drug assay, field gametocytes isolates were pre-exposed *ex vivo* for 48 h to different concentrations of the selected drugs prior to mosquito feeding. Each drug was tested in a 5-point dose-response series, including the DMSO control. The baseline infectivity of each freshly collected gametocyte isolate was systematically determined under two conditions: the whole blood DMFA (WB-DMFA) and serum replacement DMFA (SR-DMFA).

d The concentration unit varies from drug to drug. Concentrations are in nanomolar or micromolar.

Using the SCM as the base culture medium, we evaluated the infectivity of gametocytes after a 48-h *ex vivo* exposure to culture condition, which is the exposure time used for most P. falciparum drug screening assays (Fig. 1C and D). This set of experiment was performed in a series of 33 DMFAs using P. falciparum gametocyte-positive blood from 11 donors, and the gametocyte densities varied from 16 to 64 gametocytes per microliter of blood (Table 1, Table S2). The data on Fig. 1C and D show that the infectivity of *ex vivo*-cultured gametocytes is comparable to that of SR samples (oocyst prevalence, 23.9 versus 15.5, *P* = 0.1; oocyst intensity, 0.6 versus 1.5 oocyst per mosquito, *P* = 0.06).

Additional parameters, including the inactivation of the serum before medium preparation, hematocrit, and sample washing steps before DMFAs were tested to optimize *ex vivo* culture in SCM. SCM containing active complements led to the aggregation of red blood cells (RBCs) and the formation of a compact RBC monolayer that needed to be broken down during sample preparation for DMFAs (see Fig. S1A in the supplemental material). These experiments indicate that the serum inactivation facilitates sample preparation but had no impact on gametocyte infectivity. *Ex vivo* culture at 12% hematocrit decreased the infectivity but not significantly (Fig. S1B). A washing step of the *ex vivo*-cultured sample also reduced considerably the proportion of positive mosquitos.

Conventional *in vitro* drug sensitivity testing generally uses replicates within the experiment. To explore the possibility of performing the *ex vivo* drug assay with replicates and to minimize experimental variation, blood samples from different blood donors, collected the same day, were pooled and processed as a new blood sample (Fig. S1C). We performed two independent series to evaluate the blood pooling ability of gametocytes to resume the sexual life cycle in the mosquito. Overall, the oocyst prevalence from DMFAs performed with the pooled sample was comparable to that of the isolate with the highest oocyst prevalence in the pool after a 48-h *ex vivo* exposure to culture conditions (Fig. S1C).

### Establishment of the *ex vivo* transmission-blocking assay using reference antimalarial drugs and compounds in clinical development.

For the drug assay, in most cases, the baseline infectivity of gametocytes was improved by the removal of serum factor (SR sample) and *ex vivo* maintenance to culture conditions in dimethyl sulfoxide (DMSO) (control or no drug) (Fig. 1E and F; see Table S3 in the supplemental material). As shown in Fig. 1E, oocyst prevalence was significantly higher when the mosquitoes were fed with SR samples than that with WB samples (43.9 versus 27.4, *P* < 10^−4^). Similarly, the mean oocyst intensity significantly rose from 8 oocysts per mosquito to 13 oocyst per mosquito (*P* < 10^−4^) (Fig. 1F). Gametocyte infectivity was comparable from mosquitoes fed with SR and the DMSO control sample (oocyst prevalence, 48.6 versus 43.9, *P* = 0.3; oocyst intensity, 9.6 versus 13 oocyst per mosquito; *P* = 0.6) (Fig. 1E and F).

**FIG 2:**
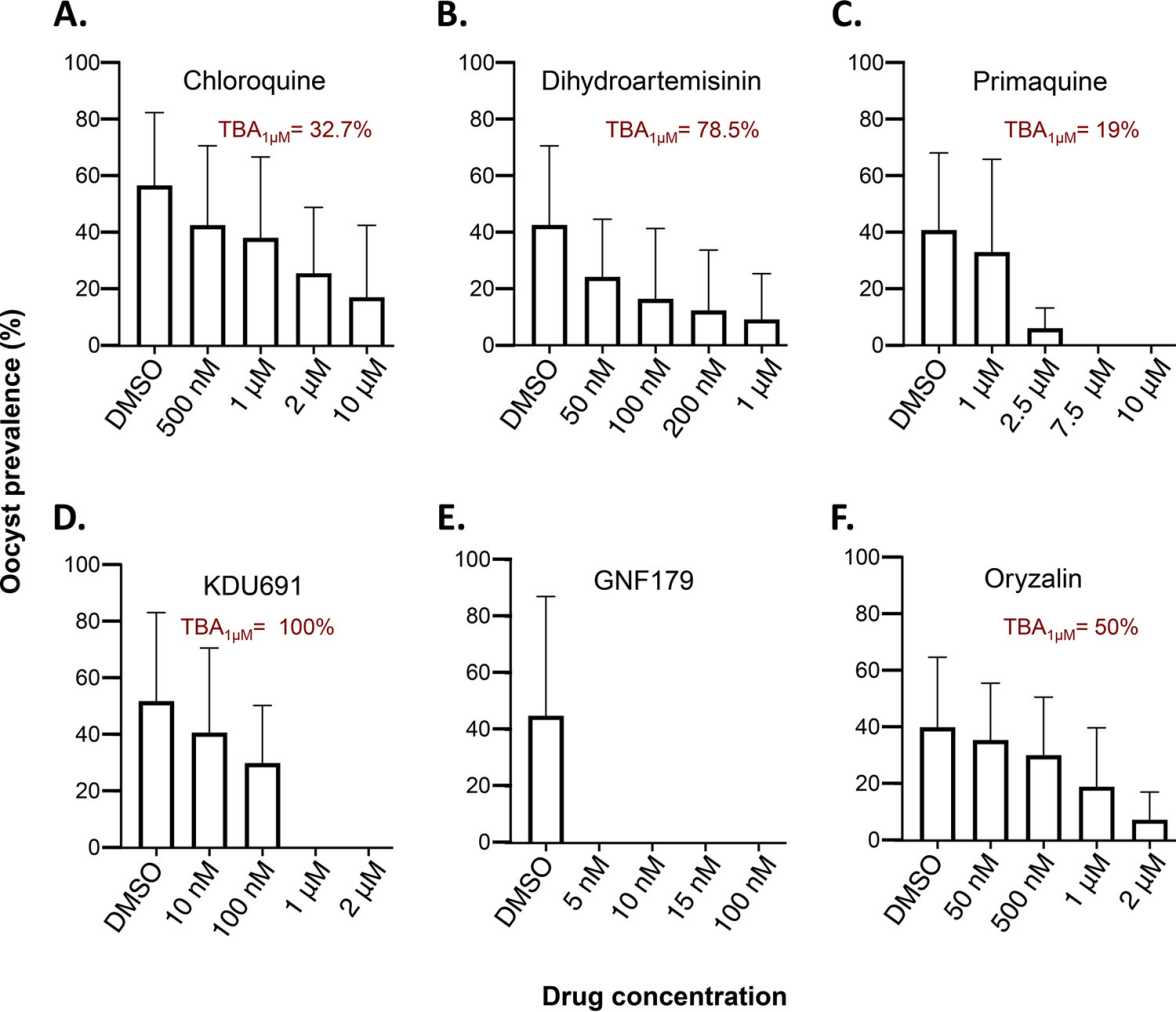
Evaluation of the activity of reference drugs on mosquito infectivity using the *ex vivo* transmission blocking assay. The histograms show the mean (± standard deviation) oocyst prevalence for the five experimental concentrations evaluated for each drug. (A) Chloroquine was ineffective to eliminate oocyst formation at 10 μM. (B) Dihydroartemisinin does not eliminate oocyst formation at 1 μM but has a transmission-blocking activity of ~78.5% at this concentration. (C) Primaquine inhibits oocyst formation by 19% at 1 μM and abolishes oocyst formation at 7.5 μM. (D) KDU691 prevented oocyst formation at 1 μM. (E) GNF179 shows high inhibition of gametocyte sexual life cycle progression in the mosquito as it abolished oocyst formation at 5 nM. (F) Oryzalin impairs oocyst formation at 1 μM with an oocyst reduction of 50%.

**FIG 3:**
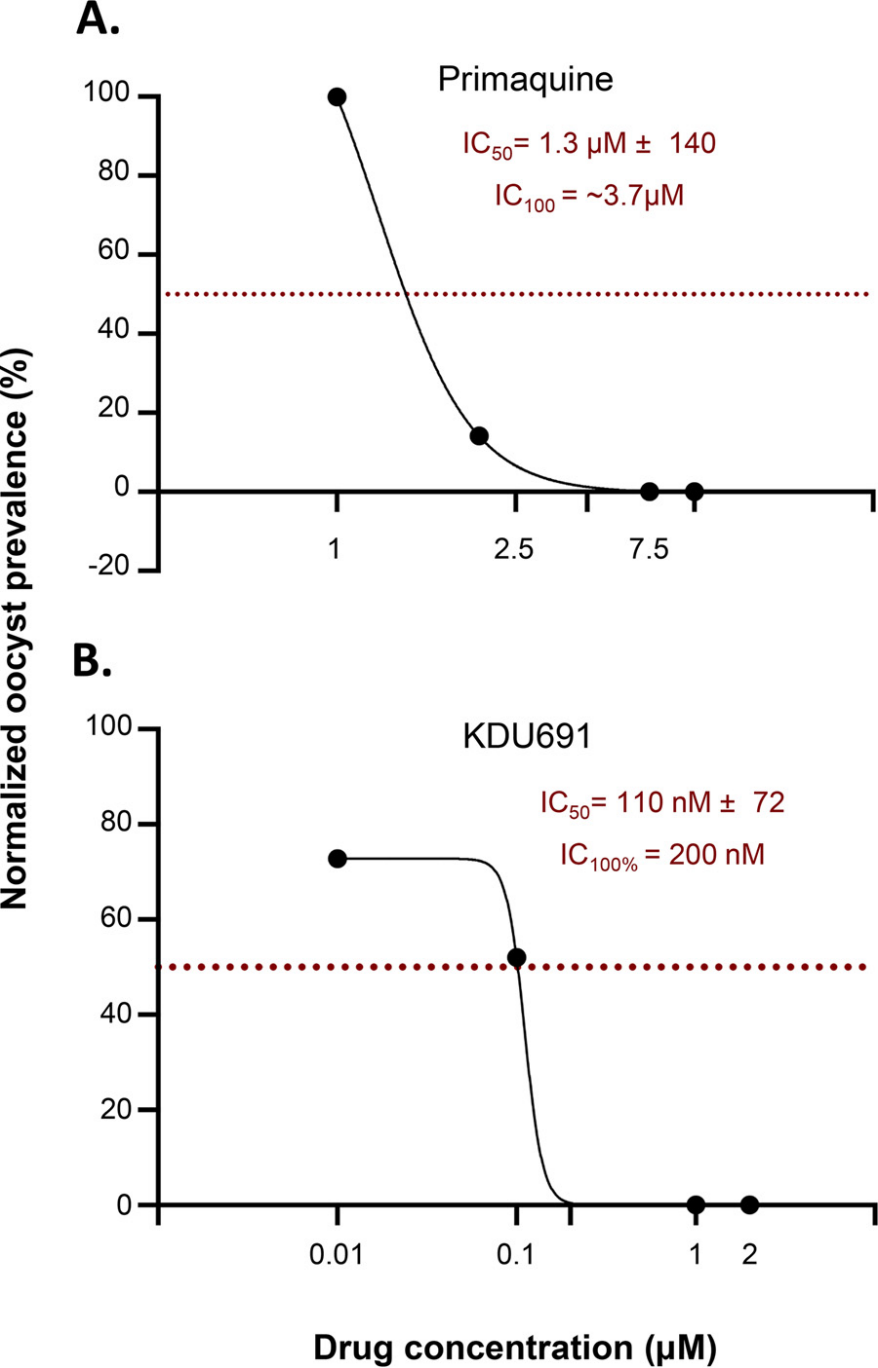
Dose response against oocyst formation. (A) Primaquine exhibited a half maximal inhibitory concentration (IC_50_) of 1.3 μM and a full maximal inhibitory concentration (IC_100_) of ~3.7 μM. (B) KDU691 exhibits an IC_50_ of 110 nM and an IC_100_ of ~200 nM. Oocyst prevalence was normalized using Prism transformation tools. First the oocyst prevalence from the DMSO control was set as 100%, and those of the various concentrations were transformed accordingly. For the plot, the three-parameter nonlinear regression model with the least-squares method to find the best fit was applied.

Chloroquine was tested on seven isolates and was ineffective at eliminating oocyst formation at 10 μM (Fig. 2A; Table 1, Table S3). Dihydroartemisinin and primaquine were both tested on 4 isolates (Table 1, Table S3). The data in Fig. 2B indicate that dihydroartemisinin does not eliminate oocyst formation at 1 μM but has a transmission-blocking activity of ~78.5% at this concentration. Primaquine inhibits oocyst formation by 19% at 1 μM (Fig. 2C) and abolishes oocyst formation at 7.5 μM. Moreover, primaquine exhibited a half maximal inhibitory concentration (IC_50_) of 1.3 μM and a full maximal inhibitory concentration (IC_100_) of ~3.7 μM (Fig. 3A).

The activity of KDU691 and GNF179 were tested on six and seven isolates, respectively (Table 1). As shown in Fig. 2D, KDU691 prevented oocyst formation at 1 μM. The analysis of the dose-response curve shows that KDU691 exhibited an IC_50_ of 110 nM and an IC_100_ of ~200 nM (Fig. 3B). The imidazolopiperazine GNF179 showed high inhibition of gametocyte sexual life cycle progression in the mosquito as it abolished oocyst formation at 5 nM (Fig. 2E).

Finally, we determined the activity of the dinitroaniline oryzalin on oocyst formation by pre-exposing seven isolates to different concentrations of the compound. Oryzalin is an herbicide targeting *Plasmodium* microtubules specifically and has been shown to have antimalarial properties against liver and asexual blood stages (33, 34). Its activity on gametocyte stages has not yet been reported. Our data indicate that oryzalin does impair oocyst formation at 1 μM (Fig. 2F) with an oocyst reduction of 50%.

## DISCUSSION

The need to characterize candidate transmission-blocking drugs thoroughly for their progress into clinical trials requires the establishment of *ex vivo* drug assays that combine the gold standard mosquito feeding assay with a reliable capture of the susceptibility of clinical gametocyte isolates circulating in regions of malaria endemicity at a given time. This study describes for the first time an optimized protocol to maintain field isolates of P. falciparum gametocytes infectious to *Anopheles* mosquito after an *ex vivo* exposure to culturing condition for 48 h. Building on this novel protocol, we then established a drug assay to evaluate the activity of compounds against mosquito infectivity, by pre-exposing field-isolated gametocytes to drugs for 48 h prior to mosquito feeding. Our assay corroborates the transmission-blocking activity of approved reference antimalarial drugs, including chloroquine, dihydroartemisinin, and primaquine. Furthermore, we demonstrate, as shown by others, that members of two novel classes of transmission-blocking compounds, the PI(4)K-inhibitor KDU691 and the imidazolopiperazine GNF179, highly inhibit clinical gametocyte isolate transmission.

Many assays were designed to evaluate the transmission-blocking activity of compounds on *Plasmodium* parasites, emphasizing the stage five gametocytes that ensure transmission to *Anopheles* mosquito (1, 22–26). However, most of these assays use laboratory-adapted isolates and fail to capture the *Plasmodium* population diversity and fail to provide a snapshot of the susceptibility of clinical isolates to candidate compounds. The mosquito feeding assay remains the gold standard tool to measure the ability of a compound to inhibit parasite development in the mosquito (28, 29). However, to date, the lack of optimized parameters to ensure gametocyte transmissibility days after blood collections from infected individuals limits the use of field-isolated gametocytes in standard drug sensitivity assays (30, 31).

Based on available protocols for the adaptation of *Plasmodium* clinical isolates and the *in vitro* production of gametocytes, we tested culture variables, such as heat-inactivated serum versus AlbuMax, whole blood versus washed blood, different starting hematocrit levels (4% and 12%), and different incubation times (24 and 48 h), to determine the optimal culture condition ensuring gametocyte viability and infectivity days after veinous sample collection (35–44). For each isolate, we determined the baseline infectivity (WB and SR sample) and the subsequent infectivity from *ex vivo* experimental culture condition (SCM, ACM, SACM). Comparing the measured infectivity variables (oocyst prevalence and oocyst intensity), we found that by setting up the culture at 4% hematocrit into a culture medium supplemented with 10% inactivated horse serum, followed by incubation in a humidified atmosphere containing 5% CO_2_ for 24 or 48 h, we can maintain gametocyte competency to infect *Anopheles* mosquitoes. The culture medium supplemented with 10% AlbuMax, a culture set up at 12% hematocrit, and a washing step of patient blood before DMFA reduced mosquito infectivity. As previously reported, our study shows that horse serum is highly suitable for field-isolated *Plasmodium* parasite culture and particularly for the production of competent gametocytes (38, 43–45). In addition, the low erythrocyte concentration (4% hematocrit) in culture appears to provide a healthy environment for the gametocytes and yopromote transmission (43, 44, 46, 47). Our experiments demonstrate that P. falciparum gametocytes, freshly collected from naturally infected individuals, can be maintained *ex vivo* for up to 48 h under specific conditions. These optimized *ex vivo* culture parameters offer an extended window to perform DMFAs, which can be helpful for transmission studies conducted in low- and middle-income countries. In addition, metabolites of field gametocyte isolates could be marked specifically during the *ex vivo* incubation time to further study biological processes relevant for transmission and infectivity.

The newly developed assay was established and validated our drug assay by pre-exposing, *ex vivo* for 48 h, collected gametocytes to drugs before the DMFA and assessed the transmission-blocking activity of chloroquine, dihydroartemisinin, primaquine, KDU691, GNF179, and oryzalin. Overall, the data align with reported activities for all the reference compounds tested in the *ex vivo* transmission-blocking assay (19, 20).

The data show that oocyst prevalence decreases with an increased concentration of chloroquine and dihydroartemisinin, but overall, the data confirm that heme detoxification in mature gametocytes is not a critical biological process (22, 48, 49). Previous clinical trials have shown that different ACT regimens could lead to gametocyte populations with different capacities to infect the *Anopheles* vector (50–52). By measuring P. falciparum gametocyte infectivity to *Anopheles* mosquitoes before and after ACT administration, Ouologuem et al. (50) showed that gametocytes from postartemether lumefantrine and post-artesunate-amodiaquine treatment are more infectious to *Anopheles* than gametocytes from post-artesunate-sulfadoxine-pyrimethamine treatment or non-drug-treated controls (50). Since artemisinin derivatives, including artesunate and artemether, are rapidly metabolized *in vivo* into dihydroartemisinin, data from this new report reinforce the hypotheses that an observed difference in infectivity following ACTs is dependent on the partner drugs. The novel drug assay combined with single-cell transcriptomics or metabolomic approaches could be used to determine the effect of partner drugs on gametocyte infectivity and to decipher the biological mechanism underlying their activity.

Primaquine, an antimalarial drug characterized by its transmission-blocking activity in the field and known to interfere with mitochondrial function, required a high concentration to eliminate oocyst formation (6, 8, 53, 54). The high primaquine concentration, also reported by other authors using SMFA, was expected, as 8-aminoquinolines need to be metabolized for activity (55, 56).

As reported earlier, the PI(4)K-inhibitor KDU691 and the imidazolopiperazine GNF179 both strongly inhibit transmission at nanomolar concentrations (17, 18).

Finally, the dinitroaniline oryzalin, which has been shown to have antimalarial properties against liver and asexual blood stages, was evaluated to determine, for the first time, its activity against mature gametocytes (33, 34). Although oocyst prevalence decreases with increased oryzalin concentration, oocyst formation could not be abolished at a micromolar concentration.

A similar approach to the drug assay could be developed to evaluate the impact of candidate transmission-blocking antibodies on clinical gametocyte isolates. Such an approach can help determine whether polymorphisms of the target protein have an impact on transmission-blocking antibodies.

Despite considerable gametocyte density variability among the samples, the baseline infectivity improved significantly when autologous plasma was replaced by nonimmune serum (WB sample versus SR- and DMSO-treated blood). It is known that factors in the blood of exposed individuals (e.g., white blood cells, antibodies, and cytokines) promote parasite killing in the mosquito blood meal or prevent fertilization in the mosquito gut (57–60). Therefore, removing these factors when setting up the DMFA experiments is key to increasing the assay’s success. Interestingly, our culture condition further improved gametocyte infectivity for most clinical isolates. Our findings suggest that the presence of morphologically matured gametocyte in a biological sample does not always reflect gametocyte transmissibility to the mosquito. The explanation of the mechanism promoted by the *ex vivo* conditions at this stage can only be speculative. Circulating stage five gametocytes could undergo further maturation after their release from the bone marrow to become fully competent, as described for *in vitro* gametocyte production, suggesting a further “subtype” of stage five gametocytes. It would be interesting to determine morphological, transcriptional, and proteomic changes of the field gametocyte population before and after *ex vivo* exposure to culture condition. In correlation with their increased infectivity, such studies could help to further characterize gametocyte subpopulations (mature versus immature and infectious versus not infectious stage V gametocytes) in endemic settings.

In conclusion, the newly developed *ex vivo* transmission-blocking assay corroborates the activity of reference drugs and compounds in clinical development. This approach will accelerate the development of new drug candidates in the drug discovery pipeline and support the global malaria eradication agenda.

## MATERIALS AND METHODS

### Ethical considerations.

The protocol was reviewed and approved by the ethical committee of the Faculty of Medicine, Pharmacy, and Dentistry, University of Sciences, Techniques and Technologies of Bamako (N˚2019/25/CE/FMPOS of 14 March 2019). Written and signed informed consents were obtained from the adult participants and from the parent or guardian of each minor participant.

### Study population and selection of gametocyte-positive blood donors.

The study was conducted in Faladié (13°8′20″N 8°20′16″W), a village located 80 km northwest of the capital Bamako in Mali. Plasmodium falciparum malaria is hyperendemic in the city, with a seasonal transmission occurring from July to December. Two cross-sectional studies were conducted from October 2019 to December 2019 and September 2020 to January 2021. During these periods, screening events were organized weekly to identify and enroll asymptomatic Plasmodium falciparum gametocyte carriers aged 6 years and above. During each screening event, a group of 45 to 100 children was invited to the Faladié health center to assess Plasmodium falciparum gametocyte carriage. Finger-prick blood smears were Giemsa stained, and the parasite counts were performed against 300 and 1,000 leucocytes for asexual parasites and gametocytes, respectively. Participants with a gametocyte density of 16 gametocytes/μL and more were clinically examined for the presence of chronic diseases and signs of acute or severe malaria. Information on any drugs taken during the preceding 2 weeks was also recorded. Asymptomatic children with a P. falciparum gametocyte density of ≥16 gametocytes/μL were selected as blood donors for direct membrane feeding assays (DMFA) scheduled for the following day at the Malaria Research and Training Center laboratory (MRTC) in Bamako.

All screened children carrying asexual *Plasmodium* stages were treated orally with pyronaridine-artesunate (Pyramax) once daily for three consecutive days (61, 62). Participants selected as gametocyte donors for the DMFA and as positive for asexual *Plasmodium* stages were treated the following day after venous blood collection. All enrolled participants were followed actively for 28 days.

### Blood sample collection.

Investigators have used various anticoagulants to collect venous blood from malaria patients, and the choice is guided by the impact of the anticoagulant on the downstream experiments. Previous studies have shown that acid citrate dextrose (ACD) does not interfere with *Taq* DNA polymerase activity and does not affect the growth of *Plasmodium* parasites when isolates are cultured immediately after blood collection (63). While heparin is known to inhibit *Taq* DNA polymerase activity, mosquito feeding experiments work best when blood is collected in a lithium heparin tube (64). Therefore, for this study, acid citrate dextrose and lithium heparin anticoagulant tubes were used for each donor venous blood collection. Blood collections on ACD and lithium heparin tubes were used for the *ex vivo* maintenance of field-isolated gametocyte experiments and the mosquito feeding assays, respectively. Both anticoagulant tubes were preheated to 37°C prior to blood collection.

For the baseline infectivity of collected gametocytes, 3 to 4 mL of blood was collected on lithium heparin tubes (VWR catalog [cat] number BD367884) and used for DMFA 45 to 90 min after veinous blood collection (64). For the *ex vivo* exposure to culture condition and downstream DMFA, 5 to 10 mL was collected on acid citrate dextrose (ACD) tubes (VWR cat number BD 364606) (63). Upon collection, all blood samples were immediately kept in the incubator at 37°C until use to avoid gametocyte activation. Care was taken to minimize sample manipulations at room temperature by using dry block heating systems whenever possible.

*Plasmodium* carriage was again assessed by microscopy using the heparinized blood sample. Blood smears were Giemsa stained, and the parasite counts were performed against 300 and 1,000 leucocytes for asexual parasites and gametocytes, respectively.

To explore the possibility of performing the *ex vivo* drug assay with replicates and of minimizing experimental variation, blood samples from different blood donors, collected the same day, were pooled and processed as a new blood sample. For the blood sample pooling experiment, an equal volume of blood collected from blood donors on the same day was mixed to create the “blood sample pool.” The sample was processed as the other blood samples (collected on heparin and ACD tubes) to assess baseline and *ex vivo* experimental condition infectivity.

### Blood sample processing for assessing the baseline infectivity of field-isolated gametocytes by DMFA.

**FIG 4:**
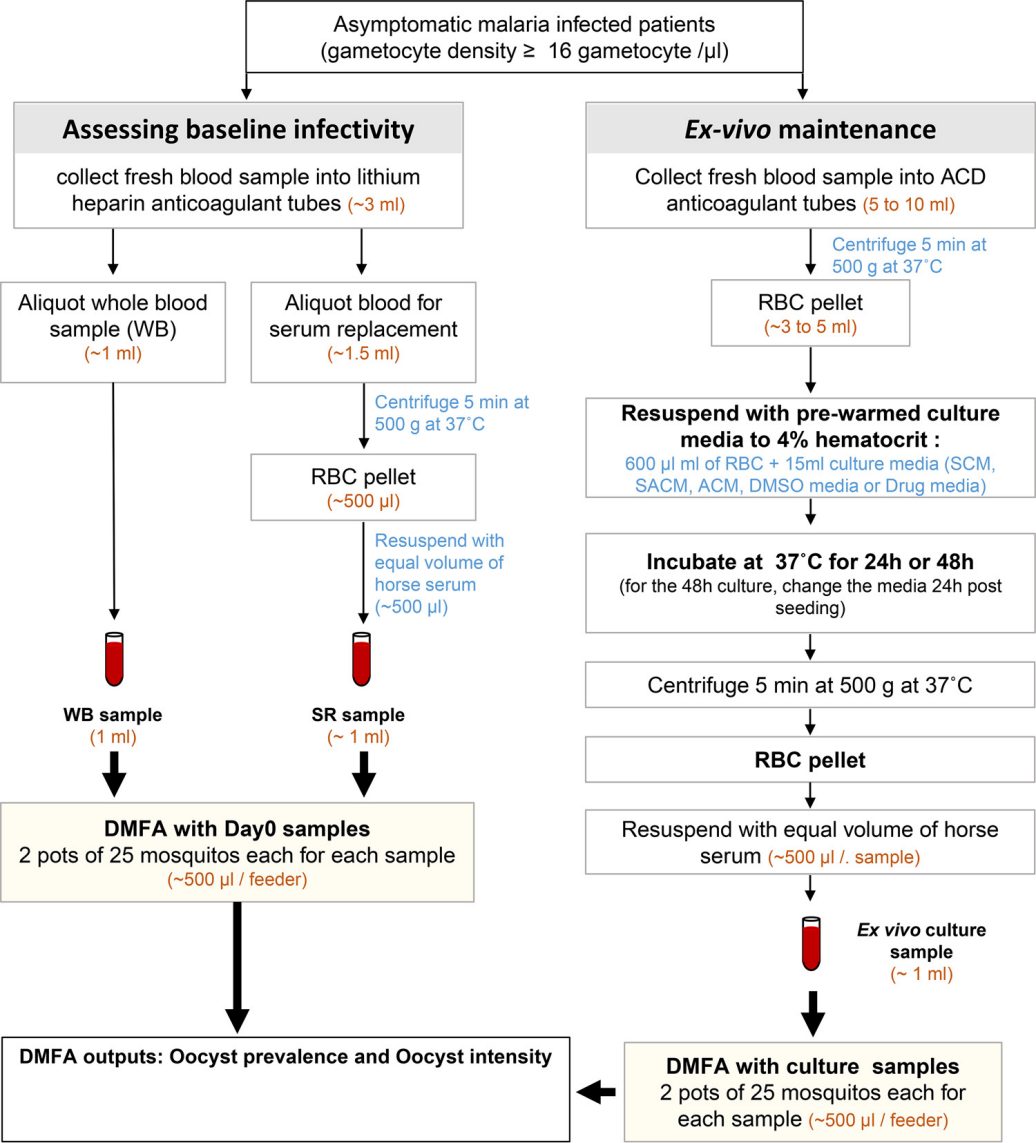
Experimental design flow chart for the *ex vivo* assessment of field-isolated gametocyte viability and infectivity. First, the baseline infectivity of field-isolated gametocytes is determined with the heparinized blood samples. The samples are treated and fed to the mosquito at 45 to 90 min after blood collection. Blood samples collected into acid citrate dextrose (ACD) anticoagulant tubes are diluted into specific culture media (serum culture medium [SCM], AlbuMax culture medium [ACM], and a serum-AlbuMax culture medium [SACM], DMSO control medium, drug-containing medium). The resulting samples are incubated at 37°C in a humidified atmosphere containing 5% CO_2_ for 24 of 48 h. Samples are subsequently prepared for direct membrane feeding assays (DMFAs). For DMFAs, infectious blood samples are fed to Anopheles coluzzii mosquitoes, and the presence (oocyst prevalence) and number of oocysts (oocyst intensity) in the gut of the mosquitoes are assessed 8 days after the infectious blood meal.

The baseline infectivity of freshly collected gametocytes was determined by using the heparinized blood samples (Fig. 4). First, 1 mL of the blood was aliquoted into a 2-mL prewarmed microcentrifuge Eppendorf tube and maintained at 37°C until the feeding experiment (whole blood DMFA [WB-DMFA]) that took place 45 to 90 min later. The remaining blood (~1.5 mL) was used for serum replacement DMFA (SR-DMFA). For SR-DMFA, the blood was centrifuged at 500 × *g* for 5 min at 37°C to collect the plasma. The RBC pellet was then resuspended with an equal volume of prewarmed horse serum (cat number MERCK H1270-500ml). The WB and SR samples were used immediately and concomitantly for DMFA (Fig. 4).

### Drug preparation.

To establish and validate the assay, we first evaluated the transmission-blocking activity of three reference compounds, with well-described antimalarials, as follows: 4-aminoquinoline chloroquine, reported to have no gametocytocidal activity against mature gametocyte stages (48); the endoperoxide dihydroartemisinin shown to have some gametocytocidal activities in SMFA (22, 48, 49); and the antimalarial drug primaquine known to block transmission in the field (6, 8, 53). To further validate our *ex vivo* assay, we then evaluated the activity of two transmission-blocking leading compounds, namely, the *Plasmodium* phosphatidylinositol-4-OH kinase (PI4K)-specific inhibitor KDU691 (18) and the imidazolopiperazine GNF179 (17).

Chloroquine (Merck, cat number C6628), dihydroartemisinin (Merck, cat number D7439), primaquine (Merck, cat number 160393), and oryzalin (MERCK, cat number 36182) were purchased from Sigma-Aldrich (Merck). KDU691 and GNF179 were kindly provided by Novartis. The drugs were prepared as a 1 M stock solution either in H_2_O (chloroquine and oryzalin) or DMSO (dihydroartemisinin, primaquine, KDU691, and GNF179) and stored at −20°C. Each drug was tested at five concentrations, including the DMSO control (untreated or no drug). The drug concentrations to be tested were selected based on published IC_50_ data (6, 8, 22, 48, 49, 53). Thus, there were differences in the concentration range tested for each drug, as follows: 500 nM to 10 μM chloroquine, 50 nM to 1 μM dihydroartemisinin, 1 μM to 10 μM primaquine, 10 nM to 2 μM KDU691, 5 nM to 100 nM GNF179, and 50 nM to 2 μM oryzalin. Drugs were freshly diluted in the culture media to achieve the required concentrations for the *ex vivo* drug assay.

### Medium for the *ex vivo* maintenance and manipulation of field-collected gametocytes.

Based on published protocols for the *in vitro* production of gametocytes, we have tested three types of culture medium to determine the optimal culture condition for the viability and infectivity of field-isolated gametocytes (35–44) (see Table S1 in the supplemental material). First, RPMI 1640 medium (Gibco, cat number 31800) was supplemented with 25 mM HEPES (Sigma, cat number H3375) and 25 mM sodium bicarbonate (Sigma, cat number S5761) and sterilized by 0.22-μm filtration with no antibiotics added to the media. For the serum culture medium (SCM) and AlbuMax culture medium (ACM), the filtered media were further supplemented with 10% heat-inactivated horse serum (MERCK, cat number H1270-500ml) and 10% AlbuMax I (Gibco, cat number 11020), respectively. For the serum-AlbuMax culture medium (SACM), the filtered medium was supplemented with both horse serum (5%) and AlbuMax I (5%).

To ensure the survival and infectivity of field-isolated gametocytes and to et the ground for subsequent drug-screening experiments, we first tested the three culture medium types (SCM, ACM, and SACM) for 24 h under culture condition (Fig. 1 and 4; Table 1). For each isolate, an aliquot of the blood sample was concomitantly diluted in SCM, SACM, and ACM and maintained under culture condition for 24 h (Fig. 4). The impact of the *ex vivo* culturing condition was assessed by comparing oocyst prevalence and oocyst intensity between the DMFA condition (Fig. 1A to D).

For the drug assay, field-isolated gametocytes were pre-exposed *ex vivo* for 48 h to different concentrations of the selected drugs prior to mosquito feeding (Fig. 1 and 4; Table 1). Each drug was tested in a 5-point dose-response series, including the DMSO control (no drug), and the oocyst prevalence was analyzed (Table 1; Fig. 1E and F, Fig. 2). The SCM was supplemented with DMSO at a final concentration of 0.02% or the drug to achieve the required concentrations for the drug assay. The isolates and the number of isolates vary between the drug tested.

The 4-aminoquinoline chloroquine was added to the SCM to final concentrations of 500 nM, 1 μM, 2 μM, and 10 μM. The endoperoxide dihydroartemisinin was added to the SCM to final concentrations of 50 nM, 100 nM, 200 nM, and 1 μM. Primaquine was added to the SCM to a final concentrations of 1 μM, 2.5 μM, 7.5 μM, and 10 μM, while PI(4)K-inhibitor KDU691 was added to final concentrations of 10 nM, 100 nM, 1 μM, and 2 μM. Imidazolopiperazine GNF179 was added to final concentrations of 5 nM, 10 nM, 15 nM, and 100 nM. Finally, dinitroaniline herbicide oryzalin was added to final concentrations of 50 nM, 500 nM, 1 μM, and 2 μM. The SCM supplemented with drugs, or DMSO, was prepared before each experiment.

Since the primary endpoint was to determine the activity of a given drug on gametocyte transmission, with the volume of blood and isolate available on the experiment day, different concentrations for a specific drug were evaluated.

### *Ex vivo* maintenance of field-isolated Plasmodium falciparum gametocytes and sample processing for DMFA.

Fresh veinous blood collected on ACD anticoagulant tubes, was used for the *ex vivo* culture experiments (Fig. 4). Blood samples were transferred into prewarmed 15-mL conical centrifuge tubes (Falcon; cat number 352099) and centrifuged at 500 × *g* for 5 min at 37°C to collect the plasma. The RBC pellets were immediately resuspended in 15 mL of prewarmed culture media at 4% or 12% hematocrit. The mixture was transferred in 75-cm^2^ culture flasks (Merck, Corning cat number CLS430641) that were incubated at 37°C in a humidified atmosphere containing 5% CO_2_ for 24 or 48 h. For the 48-h incubation experiment, the medium was replaced 24 h postseeding with the same medium composition.

To prepare samples for DMFA, samples incubated under culture conditions were transferred into prewarmed 15-mL conical falcon tubes and centrifuged at 500 × *g* for 5 min at 37°C to remove the culture medium. The RBC pellet was then resuspended with an equal volume of prewarmed horse serum (Merck; cat number H1270-500ml) and used for DMFA.

### Direct membrane feeding assays.

Direct membrane feeding assays were performed with a colony of Anopheles coluzzii originating from wild-caught mosquitoes reared in our research institution over several generations (45). The mosquitoes were from 155 to 157 generations during the first part of the study and 168 to 172 generations in the second season. Mosquitoes were reared at 25 ± 2°C with a relative humidity of 70% ± 10% (45). The larvae were fed with fish food, and the adults were fed with a 10% sugar solution or provided with human blood from the Mali blood bank to maintain the colony.

For each blood sample to be tested, 50 mosquitoes held in 2 cups of 25 female mosquitoes each were used (Fig. 4). Four- to 6-day-old females were starved for about 12 h prior to the feeding experiment and fed on 500 μL of blood per cup of mosquitoes through prewarmed membrane feeders until repletion (~10 to 15 min). DMFAs were performed using a heated circulating water bath (Model ED, v.2, DIN:12876, Julabo) and a Parafilm membrane (Bemis Company Inc., Neenah, WI). After feeding, unfed females were removed, and only fed mosquitoes were kept in the insectary. Temperature and humidity were monitored as reported previously (45). On day 8 post-DMFA, surviving mosquitoes were dissected in 0.5% mercurochrome under an optic microscope (objective, 10 and 40) for oocyst detection and quantification. The results were expressed as oocyst prevalence (the proportion of infected mosquitoes) and oocyst intensity (the mean number of oocysts per mosquito which included negative mosquitoes) (Fig. 1 and 2).

### Data analysis.

Statistical analyses were performed using GraphPad Prism (version 9) and R studio.

For the determination of the optimal culture parameters, oocyst prevalence and oocyst density were analyzed by comparing these infectivity parameters between sample treatment conditions using the Wilcoxon signed-rank test (Fig. 1) with a level of significance of 5% (*P* < 0.05).

The transmission blocking activity (Fig. 2) corresponding to the percent inhibition of a drug at a given concentration was calculated using the following formula:

$$\text{\% }\mathrm{inhibition}=100\times [1-\frac{\left(\mathrm{proportion}\;\mathrm{of}\;\mathrm{positive}\;\mathrm{mosquito}\;\mathrm{in}\;\mathrm{the}\;\mathrm{test}\;\mathrm{group}\right)}{\left(\mathrm{proportion}\;\mathrm{of}\;\mathrm{positive}\;\mathrm{mosquito}\;\mathrm{in}\;\mathrm{the}\;\mathrm{DMSO}\;\mathrm{group}\right)}]$$

IC_50_ and IC_100_ values for primaquine and KDU691 were calculated using the GraphPad Prism 9.0 software package (Fig. 3). Oocyst prevalence was normalized using Prism transformation tools. First, the oocyst prevalence from the DMSO control was set as 100%, and those of the various concentrations were transformed accordingly. For the plot, the three-parameter nonlinear regression model with the least-squares method to find the best fit was applied on Prism.
