# Supplementary material for: A Novel Ex Vivo Drug Assay for Assessing the Transmission-Blocking Activity of Compounds on Field-Isolated Plasmodium falciparum Gametocytes
Source: Antimicrob Agents Chemother. 2022 Nov 2;66(12):e01001-22. doi: 10.1128/aac.01001-22 (PMC9764978; doi:10.1128/aac.01001-22)
Supplement: Supplemental file 1 — Supplemental material. Download aac.01001-22-s0001.pdf, PDF file, 0.3 MB [file aac.01001-22-s0001.pdf]

SUPPLEMENTAL MATERIAL

Ouologuem et al. Supplementary table 1

	Catalaog number	Serum Culture Media (SCM)	Albumax Culture Media (ACM)	Serum Albumax Culture Media (SACM)
**Component**				
**RPMI 1640**	Gibco, cat# 31800	qsp	qsp	qsp
**HEPES**	SIGMA, cat# H3375	25 mM	25 mM	25 mM
**Sodium Bicarbonate**	SIGMA, cat # S5761	25 mM	25 mM	25 mM
**Horse Serum (heat inactivated)**	MERCK, cat # H1270-500ml	10%	0	5%
**Albumax**	Gibco, cat# 11020	0	10%	5%

Supplementary Table 1: Composition of the culture media tested for ex vivo preservation of field gametocytes isolates.

Ouologuem et al. Supplementary Figure 1

A.

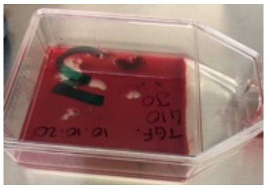

Ex vivo culture with
inactivated horse serum

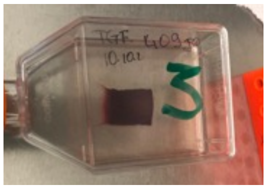

Ex-vivo culture with
complement-active horse
serum

B.

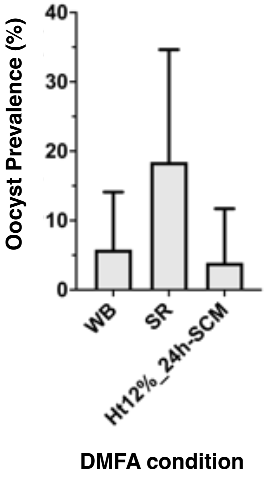

C.

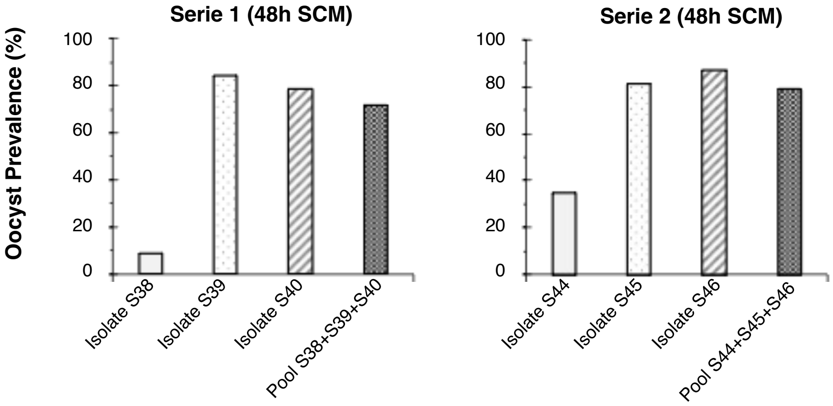

Supplementary figure 1. Additional Optimization parameters for the ex vivo maintenance of field-isolated gametocytes under culture condition in SCM

A. Testing of horse serum inactivation for parameters optimization. *Ex vivo* culture with SCM media containing active complement led to the aggregation of RBCs and the formation of a compact RBC monolayer that needed to be broken down during the DMFA sample preparation. We found that the serum inactivation facilitates sample preparation for DMFA with no impact on gametocyte infectivity. **B. Oocyst prevalence at baseline and after 24 hours *ex vivo* culture in SCM at 12%hematocrit.** *Ex vivo* maintenance of field gametocyte isolates at 12% hematocrit in SCM appear to decrease oocyst prevalence with no significance when compared to SR sample (4 vs 18.4, p=0.12). **C.** Comparison of the Oocyst prevalence from sample and the pooled sample. Blood samples from different blood donors, collected the same day, were pooled, and processed

as a new blood sample. For the pooled sample the the infectivity after maintenance in SCM under culture condition for 48 was determined. Two pooling series were performed on two independent days. Overall oocyst prevalence varied considerably between sample. The oocyst prevalence from the pooled sample DMFA seems comparable to that of the best samples used in the pool (serie 1, Pool vs isolate S39 & isolate S40; serie 2, Pool vs isolate S45 & isolate S46).
The histograms show the mean oocyst intensity of the sample.

Ouologuem et al. Supplementary table 2

					Parasite density		WB DMFA	SR DMFA	*Ex vivo* DMFA
Blood sample ID	Condition	Patient age	Patient gender	Hemoglobin (g/dl)	P.f. asexual form (Ring/µl)	P.f. sexual form (Gams/µl)	Oocyst Prevalence (%)	Oocyst Prevalence (%)	Oocyst Prevalence (%)
S01	Ht 12%, 24h culture, SCM	7	F	11.7	112	80	5.6	8.0	0
S02		7	M	10.4	3840	64	0	12.0	0
S03		7	F	8	4720	320	17.6	42.6	15.6
S04		7	M	9.6	80	96	0	11.1	0
S05	Ht 4%, 24h culture & testing 3 Media (SCM, ACM & SACM)	9	F	11.4	1960	24	5.4	8.6	
S06		8	M	9.9	1040	32	23.3	25.4	
S07		9	M	8.5	600	160	51.1	45.0	
S08		8	M	10.9	600	384	45.2	74.4	
S09		14	F	11.7	32	360	61.1	80.0	
S10		8	M	9.9	0	96	0	27.8	
S11		9	M	8.5	0	96	13.5	21.1	
S12	Ht 4%, 48h culture, SCM	7	M	10.9	0	32	38.1	51.1	64.7
S13		11	F	12.7	960	48	10.3	46.4	25.0
S14		11	F	10.9	28480	64	22.4	69.2	35.7
S15		13	F	12.9	0	16	3.8	16.7	25.0
S16		8	F	11.3	3600	24	32.6	47.9	14.7
S17		13	M	13.1	0	32	0	21.7	47.5
S18		11	M	14	0	72	5.3	44.0	80.0
S19		12	M	13	0	16	0	17.1	7.5
S20		12	F	12.4	0	24	0	10	13.2
S21		11	F	12.7	720	32	0	0	15.8
S22		8	F	11.3	0	40	12.1	16.1	11.9
Mean		9.4		10.9	904.3	41.9	15.8	31.6	
min		8		8.0	32	16	0	0	
Max		14.3		14.0	28480	384	61.1	80.0	

Supplementary Table 2: Characteristics of the blood samples used for the culture parameters optimization

Ouologuem et al. Supplementary table 3

					Parasite density		WB DMFA	SR DMFA	DMSO DMFA
Blood sample ID	Drug tested ex vivo	Patient age	Patient gender	Hemoglobin (g/dl)	P.f. asexual form (Ring/µl)	P.f. sexual form (Gams/µl)	Oocyst Prevalence (%)	Oocyst Prevalence (%)	Oocyst Prevalence (%)
S23	CQ	6	F	9.3	47564	1220	48.6	76.3	88.89
S24	CQ	13	M	12.1	32	56	5.0	20.6	42.42
S25	CQ	11	M	10.9	107	63	66.7	86.0	41.67
S26	CQ	15	F	12.9	32	48	19.2	43.8	74.36
S27	CQ	15	F	12.9	16	253	9.1	20.0	50.00
S28	CQ	6	F	9.3	0	143	50.0	55.6	17.86
S29	CQ	11	M	10.9	2641	88	33.3	44.1	81.58
S30	DHA	11	F	12.3	16159	1198	0.0	16.7	78.1
S31	DHA	9	M	10.7	3208	24	4.5	8.0	44.1
S32	DHA	11	M	11.7	538	279	0.0	4.5	38.1
S33	DHA	6	F	10.2	0	258	18.2	31.8	10.0
S34	PRQ	7	F	11.2	0	127	3.33	30.8	71.43
S35	PRQ	7	F	12.1	1419	47	7.69	41.7	43.75
S36	PRQ	7	F	12.1	0	56	0.00	16.7	5.00
S37	PRQ	11	F	12.3	16159	1198	66.67	68.4	42.86
S38	KDU691	8	M	8.1	4593	777	0.0	3.8	8.6
S39	KDU691	6	F	10	0	3888	88.2	91.3	84.4
S40	KDU691	7	M	10.6	27429	64	79.2	90.0	78.6
S41	KDU691	8	M	8.1	0	64	0.0	19.2	21.9
S42	KDU691	6	F	10	0	904	79.4	81.0	47.4
S43	KDU691	7	M	10.6	0	568	68.8	71.4	70.4
S44	GNF179	9	F	11.6	111	376	41.7	65.6	34.6
S45	GNF179	10	M	13	686	380	58.6	81.8	81.5
S46	GNF179	9	M	12.4	29373	2845	81.8	90.2	87.1
S47	GNF179	10	M	13	0	356	76.7	96.2	40.0
S48	GNF179	9	M	12.4	0	954	0.0	37.5	84.6
S49	GNF179	6	M	8.5	26819	151	0.0	20.0	6.3
S50	GNF179	7	F	11.2	4627	72	4.8	61.5	45.0
S51	ORY	12	M	12.6	1540	16	0.0	5.9	4.0
S52	ORY	6	M	11.3	2080	16	0.0	2.9	35.5
S53	ORY	7	F	10.4	836	48	21.2	50.0	53.1
S54	ORY	7	F	11	3626	822	13.8	33.3	78.4
S55	ORY	7	F	11	0	127	2.6	29.4	42.9
S56	ORY	8	F	9.9	5234	48	0.0	12.5	50.0
S57	ORY	11	F	11.6	444	24	11.5	28.1	16.0
Mean		8.6		11.1	5579.2	501.7	27.4	43.9	48.6
min		6		8	0	16	0	2.9	4
Max		15		13	47564	3888	88	96.2	88.9

Supplementary Table 3: Characteristics of the blood sample used for the ex vivo transmission-blocking assay
